# Drug-induced severe sideroblastic anemia following combined olanzapine and fluvoxamine therapy: a case report

**DOI:** 10.3389/fpsyt.2025.1637065

**Published:** 2025-09-05

**Authors:** Xinru Zhang, Anan Zhang, Jinmei Zhang, Dandan Hu

**Affiliations:** ^1^ The Third School of Clinical Medicine, Zhejiang Chinese Medical University, Hangzhou, China; ^2^ Department of Pulmonary and Critical Care Medicine, The Third Affiliated Hospital of Zhejiang Chinese Medical University, Hangzhou, China

**Keywords:** olanzapine, fluvoxamine, sideroblastic anemia, severe anemia, drug interactions

## Abstract

Olanzapine and fluvoxamine are commonly used psychotropic medications for treating anxiety and depressive disorders, particularly in cases with psychotic symptoms or treatment-resistant presentations. Although there are occasional reports of hematologic toxicity with monotherapy of these two drugs, no clear reports in the existing literature have documented severe sideroblastic anemia induced by their combination. Notably, as a potent CYP1A2 inhibitor, fluvoxamine significantly inhibits the metabolism of olanzapine, leading to elevated plasma concentrations. This pharmacokinetic synergy may exacerbate the risk of myelosuppression, although the specific mechanism remains to be elucidated. This article presents the first documented case of a 78-year-old male patient with chronic obstructive pulmonary disease (COPD) who developed severe anemia (nadir hemoglobin 37 g/L) after the combined use of olanzapine and fluvoxamine. Through systematic etiological investigation, bone marrow morphology findings, and the Naranjo Adverse Drug Reaction Probability Scale (score 9, indicating a clear association), the diagnosis was confirmed as drug-induced severe acquired sideroblastic anemia. This case underscores the importance of thoroughly evaluating blood system safety when combining psychotropic medications in elderly patients with chronic diseases, and highlights the need for enhanced dynamic monitoring to identify and intervene in potential adverse reactions at an early stage.

## 1 Introduction

Olanzapine, a thienobenzodiazepine derivative classified as a second-generation (atypical) antipsychotic, exerts its therapeutic effects primarily through antagonism of multiple neurotransmitter receptors, including 5-hydroxytryptamine (5-HT2A), dopamine D2, and histamine H1 receptors. It is effective in alleviating both positive and negative symptoms of schizophrenia and is widely used in the treatment of schizophrenia, bipolar disorder, and depression with accompanying psychotic symptoms (1). Compared to first-generation (typical) antipsychotics, olanzapine offers a superior tolerability and safety profile while maintaining therapeutic efficacy (2). Common adverse effects include weight gain, somnolence, and dizziness (3). In contrast, hematologic toxicity is relatively rare, with only a few reports in the literature suggesting that olanzapine may cause hematologic abnormalities such as granulocytopenia, leukopenia, thrombocytopenia, and pancytopenia (4–11).

Fluvoxamine is a selective serotonin reuptake inhibitor (SSRI) commonly used to treat psychiatric disorders such as obsessive-compulsive disorder, anxiety disorders, and depression (12). As it primarily targets the serotonin system and does not antagonize cholinergic, adrenergic, or histaminergic receptors, fluvoxamine has an overall favorable safety profile. The most common adverse effects are mild to moderate gastrointestinal symptoms, such as nausea and diarrhea, which are generally reversible (13). Hematologic abnormalities induced by fluvoxamine are extremely rare, with only a few reports suggesting that it may affect platelet aggregation by inhibiting serotonin uptake in platelets, thereby increasing the risk of bleeding (14–16).

Sideroblastic anemia (SA) constitutes a heterogeneous group of hematopoietic disorders characterized by pathological iron accumulation in the mitochondria of erythroid precursors, with the morphological hallmark being the presence of ringed sideroblasts (RS) in the bone marrow (≥15%). Depending on the underlying cause, SA can be classified into two types: congenital and acquired (17). Among acquired SA cases, some represent clonal myeloid disorders, such as myelodysplastic syndrome with ring sideroblasts (MDS-RS), while others are triggered by non-clonal factors, including drugs, toxins, alcohol, or nutritional deficiencies (18). Remarkably, pharmacokinetic studies show that olanzapine is primarily metabolized by the CYP1A2 enzyme, while fluvoxamine is a potent CYP1A2 inhibitor that significantly inhibits olanzapine metabolism, leading to elevated drug concentrations and an increased risk of toxicity (19).

This article presents the first documented case of a 78-year-old male patient with chronic obstructive pulmonary disease (COPD) who developed severe acquired SA (hemoglobin 37 g/L) following the combined use of olanzapine and fluvoxamine. The significant association between the adverse reaction and the drugs was confirmed through a drug timeline analysis, bone marrow morphology findings, and the Naranjo Adverse Drug Reaction Probability Scale (score 9, indicating a clear association). We conducted an in-depth discussion of its potential mechanisms, diagnostic key points, and management strategies, aiming to provide a reference for the safe combination of psychotropic medications in clinical practice.

## 2 Case presentation

### 2.1 Diagnosis and treatment of mixed anxiety and depressive disorder

On January 23, 2024, a 78-year-old Chinese male was admitted to the Department of Respiratory and Critical Care Medicine due to acute exacerbation of COPD with significant dyspnea. The patient had a history of COPD for more than ten years and had been bedridden for an extended period, receiving oxygen therapy. Upon admission, the patient developed significant emotional disturbances, including mood instability, irritability, anxiety, excessive preoccupation with his condition, and difficulty falling asleep at night. A psychiatric assessment revealed intact orientation with no hallucinations, delusions, or other psychotic symptoms. There was no suicidal ideation or aggressive behavior, and the clinical diagnosis was Mixed Anxiety and Depressive Disorder. The patient had no prior history of mood disorders and no family history of psychiatric illnesses.

On January 28, 2024, the patient commenced oral fluvoxamine 25 mg nightly, achieving initial symptomatic relief of mood disturbances. From March 1 onward, the symptoms fluctuated, accompanied by decreased treatment adherence, including resistance to healthcare personnel, non-adherence to individualized treatment plans, and nighttime agitation disturbing others’ rest. Suspecting subtherapeutic dosing as a contributing factor, the fluvoxamine dosage was increased to 50 mg nightly. Following this dose adjustment, the emotional symptoms progressively stabilized, and maintenance therapy continued for approximately 4 months.

By July 4, 2024, the patient exhibited depressive symptoms again, including emotional blunting, reduced speech and activity, social withdrawal, and decreased appetite. To optimize mood regulation, olanzapine 2.5 mg nightly was added to the ongoing fluvoxamine treatment.

### 2.2 Occurrence and management of severe anemia

Since admission, the patient’s hemoglobin (Hb) levels had remained within the normal range. However, beginning in late April 2024 (approximately 1 month after fluvoxamine dose escalation), the patient gradually developed symptoms of fatigue and dizziness. At the same time, hematologic tests showed a continuous decline in Hb and red blood cell count (RBC). By early July (after the addition of olanzapine), the decline in Hb became more pronounced, reaching a nadir of 37 g/L (reference range: 130 – 175 g/L), with the corresponding RBC count of 1.38×10¹²/L (reference range: 4.3 – 5.8×10¹²/L). During this period, the patient received a total of six blood transfusions, amounting to 9.5 units of O Rh(D)-positive leukocyte-depleted packed red blood cells, but the anemia did not show sustained improvement.

The patient had no significant bleeding history or prior hematologic disorders. Systemic etiological investigation showed no abnormalities in immunological markers (antinuclear antibodies, rheumatoid factor, immunoglobulins, blood and urine light chains). Hematological analysis revealed a reticulocyte percentage of 0.10% (reference range: 0.50 – 1.50%), indicating bone marrow hematopoietic dysfunction. On August 12, 2024, a bone marrow aspiration was performed. Immunophenotyping showed an increased proportion of nucleated red blood cells in the marrow, with mature granulocyte differentiation and no significant proliferation of primitive cell clusters. Bone marrow smear exhibited active proliferation, a decreased granulocyte-to-erythrocyte ratio, reduced cytoplasm in some immature red blood cells, and increased RS on iron staining (19%), suggesting the need to exclude MDS-RS. Bone marrow biopsy demonstrated mild active erythroid proliferation, with no significant dysplastic proliferation or increase in primitive cells. Genetic testing for SF3B1 gene mutation returned negative.

After exclusion of viral infections, neoplastic disorders, autoimmune diseases, and hereditary hematologic disorders, we observed that the progressive anemia closely correlated with the timing of fluvoxamine dosage increase and the addition of olanzapine. On September 5, 2024, considering the possibility of drug-induced anemia, we adjusted the treatment regimen by discontinuing olanzapine and reducing the fluvoxamine dosage to 25 mg nightly.

### 2.3 Outcomes and follow-up

**Figure 1:**
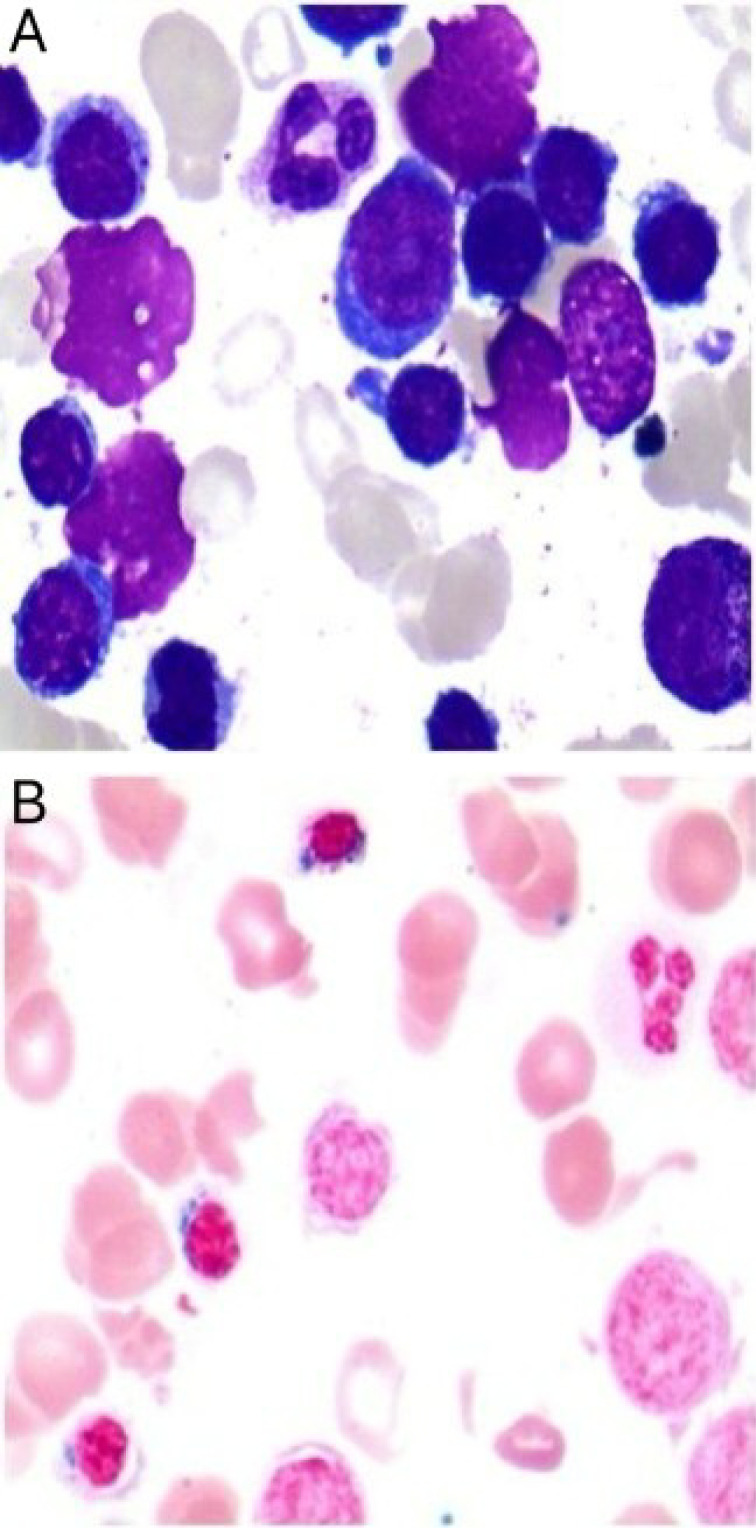
Bone marrow smear from a patient with severe acquired sideroblastic anemia. **(A)** Shows markedly active hematopoietic proliferation, a decreased granulocyte-to-erythrocyte ratio, and reduced cytoplasmic volume in some immature erythroid precursors. **(B)** Iron staining reveals an increased number of ringed sideroblasts.

**Figure 2:**
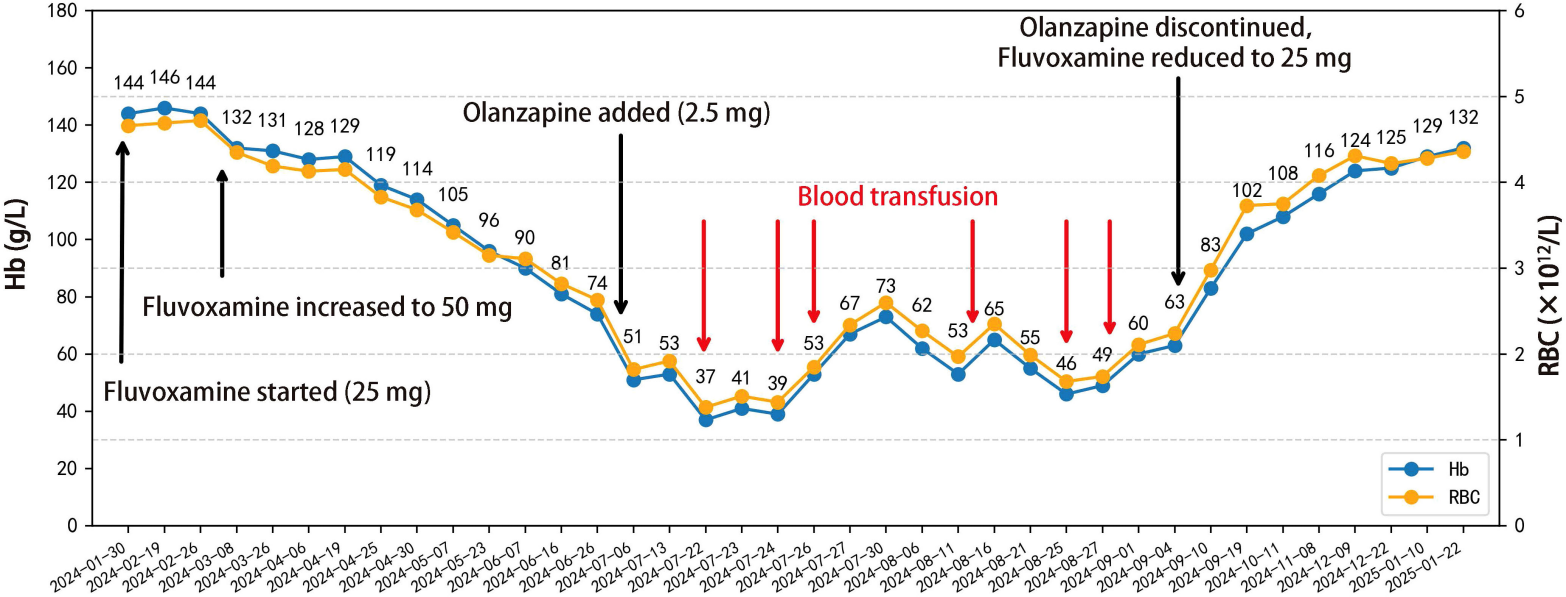
Line chart depicting the temporal changes in the patient’s hemoglobin and red blood cell levels, along with medication adjustments and the timing of blood transfusions. A total of 9.5 units of O Rh(D)-positive leukocyte-depleted packed red blood cells were transfused on July 22 (1 U), July 24 (2 U), July 26 (2 U), August 13 (1.5 U), August 25 (1.5 U), and August 28 (1.5 U), 2024.

Following therapeutic modification, the patient’s Hb exhibited a stepwise recovery, rising from 63 g/L to 102 g/L within two weeks and nearly returning to the normal range within three months. Symptoms such as fatigue and dizziness also alleviated simultaneously. During the five-month follow-up period, the patient’s routine blood tests remained stable within the normal range, with no recurrence of anemia or need for further blood transfusions or other targeted treatments. The patient’s bone marrow morphology results are shown in **Figure 1**, and the dynamic changes in Hb and RBC are shown in **Figure 2**. The patient’s inpatient medication list is shown in **Table 1**, changes in liver function indicators are shown in **Table 2**, and changes in renal function indicators are shown in **Table 3**.

**Table 1:** List of medications administered during the patient’s hospitalization.

Indication	Medication	Dose	Frequency
Asthma, COPD, and allergy-related diseases	Montelukast sodium	10 mg	Once daily at bedtime
	Loratadine	10 mg	Once daily at bedtime
	Theophylline sustained-release	100 mg	Twice daily (08:00, 16:00)
Anti-inflammatory, immunosuppressive	Methylprednisolone	4 mg	Once daily
Mucolytic, expectorant	Ambroxol	30 mg	Twice daily (08:00, 16:00)
Gastroesophageal reflux disease, peptic ulcer	Pantoprazole sodium	40 mg	Once daily before meals
Dyslipidemia and secondary prevention of atherosclerotic cardiovascular disease	Atorvastatin calcium	20 mg	Once daily at bedtime
Secondary prevention of coronary heart disease, angina pectoris, and cerebrovascular disease	Aspirin enteric-coated	100 mg	Once daily
	Isosorbide mononitrate	20 mg	Twice daily (08:00, 16:00)
	Trimetazidine sustained-release	35 mg	Twice daily (08:00, 16:00)
	Bisoprolol	2.5 mg	Once daily
Cardiac edema, related fluid retention	Furosemide	20 mg	Twice daily (08:00, 16:00)
	Spironolactone	20 mg	Once daily
Peripheral neuropathy, vitamin B12 deficiency	Mecobalamin	0.5 mg	Three times daily
Psychiatric, emotional disorders	Fluvoxamine	25/50 mg	Once daily at bedtime
	Olanzapine	2.5 mg	Once daily at bedtime

**Table 2:** Liver function parameters during the patient’s hospitalization.

Date	ALT(U/L)	AST(U/L)	ALP(U/L)	GGT(U/L)	TBil(µmol/L)	Alb(g/L)
2024/01/23	18.3	17.9	99.8	22.1	13.88	42.5
2024/02/19	24.1	21.9	78.6	29.3	9.00	33.5
2024/03/01	34.7	23	86.7	58.8	17.10	32
2024/03/30	25.6	19.4	99.9	25.3	14.1	29.3
2024/04/30	38.4	18.1	87.9	25.0	12.80	32.3
2024/05/23	19.0	10.7	96.1	25.3	6.60	32.1
2024/06/16	10.4	8.7	92.0	19.0	6.28	34.5
2024/07/22	9.4	8.1	73.4	20.1	12.60	38.4
2024/08/06	5.8	7.4	86.5	18.8	12.50	38.4
2024/09/19	9.1	12.5	94.6	20.5	10.90	42.6
2024/10/11	12.4	15.1	78.3	18.3	6.50	34.9
2024/11/08	10.8	14.5	86.3	19.8	4.80	34.3
2024/12/22	22.3	20.1	76.1	16.5	6.06	33.8
2025/01/22	38.1	27.8	71.9	19.5	19.14	37.6

**Table 3:** Renal function parameters during the patient’s hospitalization.

Date	Scr(µmol/L)	BUN(mmol/L)	eGFR	K^+^(mmol/L)	Na^+^(mmol/L)
2024/01/23	78.5	6.54	82.17	4.25	137.5
2024/02/19	70.7	10.46	85.73	4.75	136.1
2024/03/01	58.6	10.29	92.59	3.90	135.6
2024/03/30	68.1	13.43	87.00	5.07	143.1
2024/04/30	53.7	8.41	95.86	3.64	131.3
2024/05/23	68.6	12.18	86.85	4.08	129.7
2024/06/16	57.8	9.35	92.92	4.17	134.4
2024/07/22	81.5	12.06	79.44	4.40	123.4
2024/08/06	64.7	11.15	88.63	4.78	125.3
2024/09/19	78.8	9.72	81.66	4.09	133.3
2024/10/11	60.9	9.55	90.75	3.97	139.7
2024/11/08	54.7	6.98	94.79	3.89	142.7
2024/12/22	68.4	9.04	86.40	3.66	138.1
2025/01/22	67.7	10.15	86.71	3.74	143.1

## 3 Discussion

### 3.1 Diagnosis of severe acquired sideroblastic anemia

This study reports a case of a 78-year-old male patient with COPD who was diagnosed with Mixed Anxiety and Depressive Disorder during hospitalization. The patient developed severe anemia (nadir Hb 37 g/L) following combined administration of olanzapine (2.5 mg nightly) and fluvoxamine (50 mg nightly). Despite receiving six blood transfusions, the anemia did not improve. After exclusion of alternative etiologies, drug-induced anemia was suspected, and the treatment regimen was adjusted: olanzapine was discontinued, and fluvoxamine was reduced to 25 mg nightly. Subsequently, the patient’s Hb rose stepwise and returned to the normal range within three months, with sustained stability during the five-month follow-up.

In this case, bone marrow iron staining revealed an RS proportion of 19%, consistent with the morphological features of SA. According to the 2022 International Consensus Classification of myeloid neoplasms and acute leukemia (20), a diagnosis of SF3B1-mutant myelodysplastic syndrome can be made when an SF3B1 mutation is present and RSs comprise ≥5% of erythroid precursors. In the absence of an SF3B1 mutation, a diagnosis of MDS-RS requires an RS proportion ≥15%, with secondary causes excluded. Although the RS proportion in this patient exceeded 15%, SF3B1 testing was negative, and there was no significant dysplasia or increase in blasts in the bone marrow, thus not fulfilling the morphological or molecular diagnostic criteria for MDS-RS. Furthermore, the patient had a clear history of suspected drug-induced etiology (olanzapine combined with fluvoxamine). After the drug withdrawal intervention, the Hb rapidly rose from 63 g/L to 102 g/L within two weeks, normalized within three months, and no further transfusions were required, demonstrating a clear temporal correlation and reversibility, which is inconsistent with the natural course of MDS-RS as a clonal marrow disease.

Considering the patient’s RS ≥ 15%, SF3B1 negativity, absence of clonal characteristics in the hematopoietic system, nadir Hb level of 37 g/L, coupled with a clear drug-related cause and significant improvement following drug withdrawal, the diagnosis of severe acquired sideroblastic anemia induced by the combined use of olanzapine and fluvoxamine was established. The Naranjo Adverse Drug Reaction Probability Scale score of 9 further supports this conclusion.

### 3.2 Drug interaction between olanzapine and fluvoxamine

It is noteworthy that the patient developed progressive severe anemia following the addition of olanzapine to fluvoxamine therapy. Although such a phenomenon has been rarely reported in the literature, studies have demonstrated significant drug interactions between olanzapine and fluvoxamine (21–26). Based on clinical therapeutic drug monitoring data, Weigmann et al. (27) analyzed the pharmacokinetic changes resulting from the combined use of olanzapine and fluvoxamine. They found that fluvoxamine, a potent CYP1A2 inhibitor, significantly inhibits the metabolism of olanzapine, leading to an average 2.3-fold increase in its plasma concentration, with some patients exhibiting increases of more than 4-fold, approaching the potential toxicity threshold (>100 ng/mL).

In this case, after discontinuing olanzapine and reducing the fluvoxamine dosage, the anemia improved rapidly, with Hb levels significantly rising within a short period. Combining the temporal sequence, laboratory changes, and previous pharmacokinetic studies, this suggests that the adverse reaction may be closely related to olanzapine accumulation caused by the drug combination.

### 3.3 Possible role of fluvoxamine monotherapy in this case of severe acquired sideroblastic anemia

While the above evidence points to the drug interaction as the primary driver, the possibility that fluvoxamine monotherapy contributed to the development of anemia merits consideration. Given that the patient’s Hb levels had already declined before the initiation of olanzapine therapy, we evaluated the potential cumulative effects of fluvoxamine alone. However, several factors suggest its likelihood of being the primary cause of severe acquired SA is low. Firstly, no published cases to date have implicated fluvoxamine monotherapy in SA, and in this case, the degree of Hb reduction during the monotherapy phase did not reach the diagnostic threshold for severe anemia. Secondly, the precipitous drop in Hb to 37 g/L closely coincided with olanzapine initiation, and the pathological finding of a 19% ring sideroblast proportion on bone marrow iron staining also occurred during the combination therapy phase. Moreover, after discontinuation of olanzapine, which was accompanied by a dose reduction but not cessation of fluvoxamine, the patient’s Hb rose rapidly by 39 g/L within two weeks, suggesting that olanzapine was the main pathogenic factor.

Nonetheless, a contributory role of fluvoxamine monotherapy in the development of anemia cannot be entirely excluded. Epidemiological studies have indicated that SSRIs may be associated with secondary anemia linked to bleeding tendencies. In a prospective cohort study in Paris involving 6,854 participants, Vulser et al. (28) found that both depressive states and SSRI use were significantly associated with lower Hb levels. Additional studies have shown that SSRI users generally have lower Hb concentrations compared to non-users, possibly due to inhibition of platelet serotonin uptake, impairment of platelet aggregation, and prolonged bleeding time, which may result in chronic occult blood loss and secondary anemia. Such anemia differs fundamentally in pathophysiology from drug-induced hematopoietic failure in SA. Although fluvoxamine was not specifically evaluated in that study, as an SSRI it warrants caution in elderly patients or those with chronic comorbidities. In this case, fluvoxamine may have contributed to the early phase of anemia, but this remains unsupported by direct evidence.

### 3.4 Potential mechanisms underlying drug-induced acquired sideroblastic anemia

#### 3.4.1 Mitochondrial dysfunction

Mitochondrial dysfunction is one of the core mechanisms underlying the pathogenesis of SA. Normal iron metabolism, efficient heme synthesis, and oxidative phosphorylation in erythroid precursor cells all depend on fully functional mitochondria. When mitochondrial function is disrupted, heme synthesis is hindered, preventing iron from being properly incorporated into the porphyrin ring. As a result, iron accumulates abnormally in the mitochondria of erythroid precursors, forming RS, which in turn interferes with erythrocyte maturation and release (29). Rodriguez-Sevilla et al. (30) summarized that the common features of acquired SA include impaired heme synthesis within the mitochondria, disrupted iron-sulfur cluster synthesis, and damaged mitochondrial protein synthesis. These factors collectively induce mitochondrial iron overload, triggering cytotoxic stress responses, which then suppress erythroid development.

Recent studies have shown that the accumulation of olanzapine in the body can lead to mitochondrial dysfunction in erythroid precursor cells, which in turn affects heme synthesis and erythropoiesis (31). Specifically, it can damage the mitochondrial structure, induce mitochondrial fragmentation, inhibit mitochondrial autophagy, and disrupt mitochondrial homeostasis. In addition, olanzapine has been found to downregulate the gene expression of mitochondrial respiratory chain enzymes and reduce their activity, thereby impairing cellular energy metabolism and indirectly suppressing erythropoiesis (32).

Similarly, clinical reports have indicated that certain mitotoxic drugs, such as chloramphenicol and linezolid, can induce reversible sideroblast formation and anemia by inhibiting mitochondrial protein synthesis, further confirming that drug interference with mitochondrial function may drive acquired SA (30, 33). Therefore, it is speculated that in this case, fluvoxamine inhibited the CYP1A2 enzyme, leading to excessive accumulation of olanzapine in the body. This, in turn, impaired hematopoietic precursor mitochondrial function, thereby inducing acquired SA.

#### 3.4.2 Oxidative stress

Elevated plasma concentrations of olanzapine can induce excessive oxidative stress, leading to damage of erythroid precursor cells and the development of erythropoietic disorders. Previous studies have shown that olanzapine increases oxidative stress and impairs mitochondrial function, thereby disrupting the normal differentiation of erythroid precursors. Boz et al. (31), using a mouse hypothalamic neuron model, reported that olanzapine significantly elevated reactive oxygen species (ROS) levels and downregulated the expression of key antioxidant enzymes, including glutathione reductase (GSR), catalase (CAT), and glutathione peroxidase (GPX1). This disruption of redox homeostasis ultimately triggered cellular apoptosis. These findings are consistent with those of Martin et al. (29), who demonstrated in a superoxide dismutase 2 (SOD2)-deficient animal model that the absence of SOD2 in erythroid precursor cells led to the excessive accumulation of mitochondrial superoxide anions. This oxidative imbalance induced iron metabolism dysregulation and mitochondrial iron deposition, promoting the formation of RS and ultimately resulting in SA.

Kramar et al. (32) further compared the oxidative stress responses of olanzapine and aripiprazole in hepatocytes and found that the olanzapine treatment group had significantly increased ROS levels, decreased glutathione content, and a marked reduction in antioxidant responses, suggesting that olanzapine disrupted redox balance and caused sustained mitochondrial damage. More importantly, the study also showed that olanzapine activates multiple oxidative stress signaling pathways, including members of the mitogen-activated protein kinase (MAPK) family (such as p38, JNK, and ERK), and signal transducer and activator of transcription 3 (STAT3). It also enhanced the activity of caspase-3/7 and caspase-9, activating mitochondrial-mediated programmed cell death. These changes may directly induce apoptosis or dysfunction of erythroid precursor cells, thereby inhibiting erythropoiesis.

In summary, the accumulation of olanzapine in the context of fluvoxamine-mediated metabolic inhibition may exacerbate oxidative stress, damaging erythroid precursor cells and thereby inducing erythropoietic suppression and SA.

#### 3.4.3 Endoplasmic reticulum stress

The endoplasmic reticulum (ER) is a pivotal organelle responsible for protein synthesis and folding, and its proper function is particularly critical for hemoglobin synthesis in erythroid cells (34, 35). The accumulation of unfolded or misfolded proteins within the ER lumen triggers ER stress and activates the unfolded protein response, which is mediated primarily through the ATF6, IRE1, and PERK pathways. Persistent ER stress can ultimately lead to apoptosis (36, 37). Previous studies have demonstrated that ER stress plays an important role in ineffective erythropoiesis. Hyperactivation of ER stress can induce erythropoietic defects caused by DNA methyltransferase 1 deficiency via the P53–Caspase3 pathway (38). Additionally, ER stress can impair erythropoiesis by suppressing erythropoietin production, thereby contributing to anemia (39). Another study (35) reported that CD44 deficiency results in reactive oxygen species accumulation and sustained activation of the PERK/eIF2α/ATF4/CHOP signaling pathway, leading to protein folding defects and disruption of proteostasis in erythroid cells. This, in turn, inhibits terminal differentiation and enucleation, culminating in ineffective erythropoiesis. Furthermore, the study suggested that the CD44–hyaluronic acid axis is associated with iron endocytosis, and that this stress state may further impair iron utilization, thereby exacerbating erythroid defects.

In recent years, multiple studies have shown that olanzapine can induce pronounced ER stress in various cell types. For example, in pancreatic β-cells, olanzapine triggers significant ER stress and blocks PERK-mediated protective translational attenuation, leading to the continuous accumulation of misfolded proteins and ultimately apoptosis (40). In hypothalamic astrocytes, olanzapine likewise induces ER stress and promotes apoptosis (41). More recent findings (42) have demonstrated that olanzapine activates the PERK–CHOP signaling pathway in adipocytes, inducing marked ER stress and disrupting key functions such as lipid metabolism and inflammatory cytokine secretion, suggesting that its effects may extend to metabolically relevant cell types.

Taken together, olanzapine possesses the potential to induce ER stress and promote apoptosis in multiple cell types, while erythroid precursor cells similarly depend on efficient protein folding and proteostasis to accomplish hemoglobin synthesis and terminal differentiation. We therefore hypothesize that, under certain conditions, if olanzapine were to trigger a comparable ER stress response within the erythroid system, it could disrupt protein folding and iron metabolism, suppress erythroid maturation, and lead to ineffective hematopoiesis, thereby precipitating or aggravating anemia. Notably, when olanzapine is co-administered with fluvoxamine, its plasma concentration can rise markedly, and the resulting ER stress response may be further amplified, thus exacerbating erythroid dysfunction and increasing both the risk and severity of anemia.

#### 3.4.4 Inhibition of erythroid progenitor cell proliferation and apoptosis

Olanzapine and its metabolites may directly exert toxicity on bone marrow erythroid progenitor cells, inhibiting their proliferation or inducing apoptosis, thereby reducing erythropoiesis. Animal studies have shown that olanzapine, through itself or its unstable metabolites, can directly affect hematopoietic tissues. For example, in a mouse pup model, exposure to olanzapine during the lactation period significantly reduced peripheral blood neutrophil counts, suggesting that it may directly inhibit bone marrow hematopoietic cells (43). Since both erythrocytes and granulocytes are derived from the bone marrow stem cell pool, this direct toxicity may also impair the proliferation and survival of erythroid progenitor cells.

Despite being rare in clinical practice, case reports of olanzapine-induced pancytopenia have been documented (5–7, 10, 44). While the underlying mechanisms remain incompletely elucidated, some studies propose similarities with clozapine: reactive metabolites generated during metabolism (such as nitroso ions) covalently bind to critical proteins within hematopoietic cells, eliciting cytotoxic effects or immune-mediated myelosuppression. Notably, most of these patients gradually recover their blood cell counts after discontinuation of the drug, indicating that olanzapine-induced myelosuppression is reversible. Furthermore, emerging evidence suggests that olanzapine may induce granulocytopenia or neutropenia through mechanisms encompassing immune-mediated cytotoxicity, oxidative stress, and direct metabolite-induced damage to hematopoietic progenitors (45).

### 3.5 Limitations

This study has the following limitations: Due to clinical constraints, plasma concentrations of olanzapine and fluvoxamine were not monitored. Although therapeutic drug monitoring (TDM) is not routinely implemented for these agents in clinical practice, obtaining pharmacokinetic data could directly confirm the *in vivo* accumulation of olanzapine. Future studies could incorporate TDM in high-risk patients receiving combined olanzapine–fluvoxamine therapy to define the exposure thresholds associated with hematologic toxicity, thereby providing stronger evidence to guide safe clinical use.

## 4 Conclusion

This article reports the first documented rare case of severe acquired SA induced by combined olanzapine-fluvoxamine therapy. Based on bone marrow morphological characteristics, SF3B1 negativity, temporal drug exposure correlation, and rapid hematologic recovery post-cessation, clonal myeloid disorders such as MDS-RS were excluded, with drug-induced erythropoietic dysfunction concomitant with secondary iron metabolism abnormalities being established. Mechanistically, fluvoxamine-mediated metabolic inhibition leads to olanzapine accumulation, potentially interfering with erythropoiesis through multiple pathways, including mitochondrial dysfunction (impaired heme synthesis, disturbed iron-sulfur cluster metabolism), oxidative stress (elevated ROS levels, imbalance in the antioxidant system), endoplasmic reticulum stress (disrupted protein folding and proteostasis), and erythroid progenitor apoptosis. Additionally, as an SSRI agent, fluvoxamine may exacerbate erythroid suppression through platelet dysfunction, occult hemorrhage, and chronic inflammatory processes, thereby amplifying the myelotoxicity of olanzapine.

This case underscores that in clinical practice, when implementing psychotropic polypharmacy, the impact of pharmacokinetic interactions on plasma drug concentrations and potential toxicity warrants rigorous assessment. Particularly in elderly patients and those with chronic comorbidities, therapy should be initiated at low doses with serial monitoring of plasma concentrations and hematologic parameters to optimize treatment safety.

## Data Availability

The original contributions presented in the study are included in the article/supplementary material. Further inquiries can be directed to the corresponding author.
